# Combined Exposure to Simulated Microgravity and Acute or Chronic Radiation Reduces Neuronal Network Integrity and Survival

**DOI:** 10.1371/journal.pone.0155260

**Published:** 2016-05-20

**Authors:** Giuseppe Pani, Mieke Verslegers, Roel Quintens, Nada Samari, Louis de Saint-Georges, Patrick van Oostveldt, Sarah Baatout, Mohammed Abderrafi Benotmane

**Affiliations:** 1 Radiobiology Unit, Laboratory of Molecular and Cellular Biology, Institute for Environment, Health and Safety, Belgian Nuclear Research Centre, SCK•CEN, Mol, Belgium; 2 Cell Systems and Imaging Research Group (CSI), Department of Molecular Biotechnology, Ghent University, Ghent, Belgium; 3 Laboratory of Membrane Biochemistry and Applied Nutrition, Department of Pharmacology and Bio-molecular Sciences (DiSFeB), Università degli Studi di Milano, Milano, Italy; University of Louisville, UNITED STATES

## Abstract

During orbital or interplanetary space flights, astronauts are exposed to cosmic radiations and microgravity. However, most earth-based studies on the potential health risks of space conditions have investigated the effects of these two conditions separately. This study aimed at assessing the combined effect of radiation exposure and microgravity on neuronal morphology and survival *in vitro*. In particular, we investigated the effects of simulated microgravity after acute (X-rays) or during chronic (Californium-252) exposure to ionizing radiation using mouse mature neuron cultures. Acute exposure to low (0.1 Gy) doses of X-rays caused a delay in neurite outgrowth and a reduction in soma size, while only the high dose impaired neuronal survival. Of interest, the strongest effect on neuronal morphology and survival was evident in cells exposed to microgravity and in particular in cells exposed to both microgravity and radiation. Removal of neurons from simulated microgravity for a period of 24 h was not sufficient to recover neurite length, whereas the soma size showed a clear re-adaptation to normal ground conditions. Genome-wide gene expression analysis confirmed a modulation of genes involved in neurite extension, cell survival and synaptic communication, suggesting that these changes might be responsible for the observed morphological effects. In general, the observed synergistic changes in neuronal network integrity and cell survival induced by simulated space conditions might help to better evaluate the astronaut's health risks and underline the importance of investigating the central nervous system and long-term cognition during and after a space flight.

## Introduction

Cosmic radiations and microgravity, combined with heavy workload, confinement and psychological stress, are the main stressors that affect the astronaut's health during space flight. The continuous free fall condition aboard the International Space Station (ISS) or spacecrafts orbiting earth, known as microgravity, induces physiological and metabolic changes in the human body as well as at the cellular level [1–9].

Cosmic radiations are a heterogeneous pool of ionizing radiations (protons, α-particles and HZE particles) with a wide range of charges and energies [10], mainly produced by galactic cosmic rays and solar particle events [11]. Dosimetry performed with TLD-100 dosimeters of 19 astronauts aboard the ISS for about 180 days allowed to determine a total dose of 28.9±4.9 mGy and a dose of 71.9±12 mSv as prediction of the effective dose [12]. These analysis allowed to estimate the average dose rate of cosmic radiation, being in the order of 0.4 mSv or 0.16 mGy per day [12], while during a travel to Mars an equivalent dose rate of about 1.8 mSv per day (0.48 mGy with QF 3.82) has been estimated [13]. Furthermore, secondary neutrons that originate from the interaction of primary particles with the spacecraft shield are thought to contribute to the overall radiation exposure [11, 14, 15]. Like microgravity, chronic exposure to space radiation can also induce a range of physiological defects [16–25]

Neuroplasticity is an ability of the adult brain to remodel the network by contracting and re-extending neurites and to reorganize connections between neurons in response to environmental changes [17]. The neurite outgrowth motility involves several cytoskeletal proteins, in particular stable microtubules along the extensions, actin and integrins at the periphery of the outgrowth cone [21]. It is relevant to mention that simulated microgravity alters morphology, cytoskeletal organization and cellular motility in *in vitro* primary neurons [26] as well as related cytoskeleton protein expression in rat hypothalamus [27, 28] and mouse hippocampus and hypothalamus [29, 30]. Furthermore, analysis of brain sections of rodents exposed to real or simulated microgravity highlighted changes in proteins involved in oxidative stress, Parkinson and Alzheimer diseases, response to environmental stresses, neurotransmitter turnover, neurotransmitter release, glucose metabolism and apoptosis [27–31]. Nowadays, many uncertainties still exist about space radiation risks to the central nervous system (reviewed in [32] and [33]). Investigations on neurons exposed to ionizing radiation have reported effects on spine number and synapse clusters after 14 days of high dose exposure [34], as well as neuronal cell death of maturing neurons after exposure to low doses (0.2 Gy) of X-rays [35]. Moreover, *in vivo* studies indicated that low and high doses of HZE particles, such as Fe and Ar, are capable of producing morphological, neurochemical and behavioral alterations, including apoptosis, oxidative stress, cognitive dysfunction, neurogenesis inhibition and memory impairment [36–42], while a correlation could be observed between neurodegenerative diseases and heavy ion exposure in a mouse model [43]. Moreover, mice acutely irradiated with O and Ti charged particles showed cognitive decrement, persistent reduction in dendritic arborization and spine density, and altered synaptic integrity 6 weeks after exposure [44]. Additionally, neurological disorders such as mental fatigability, reduced learning ability and increase of irritability have been reported in populations exposed to chronic low dose of ionizing radiation [19, 45].

Although consequences of exposure to either microgravity or irradiation are well documented, only few experiments have been performed combining both conditions. For example, a decreased apoptosis could be demonstrated in fetal fibroblasts cultured for 24 h under simulated microgravity after exposure to moderate (0.5 Gy) and high (1 Gy) doses of X-rays [46]. In addition, a delay in DNA repair kinetics accompanied by an increased apoptosis was observed in peripheral lymphocytes exposed to simulated gravity and high doses of gamma irradiation, as compared to cells that were only exposed to irradiation [47]. Interestingly, in mouse fetal fibroblasts exposed to simulated microgravity and chronic radiation (neutron source), a gene expression analysis concomitantly revealed changes in genes related to cytoskeletal rearrangements, cell motility, oxidative stress, cell signaling and the cell cycle [18]. To the best of our knowledge, the simultaneous effect of both space conditions on nervous system functionality and remodeling has not yet been investigated. Therefore, in this study, we investigated the combined effect of microgravity and irradiation (acute X-ray and chronic neutron exposure) on *in vitro* well-connected primary mouse cortical neurons, in order to better comprehend the consequences of space conditions to the central nervous system through neuronal network evaluation. Conscious about limitations of 2D *in vitro* neuron cultures used as an adult CNS model, we previously investigated neuronal connectivity in our *in vitro* cultures as a hallmark of an adult neuronal network. This showed that in the *in vitro* model, the stage of reasonable connectivity [48] was reached at about the 10^th^ day of culture (10^th^ day *in vitro*, DIV) [49]. At this stage, mature neurons are characterized by spontaneous burst activity, inhibitory activity of GABA and glycine neurotransmitters [50, 51], and increased resistance to exogenous stressors (compounds, environmental factors and infections) [52, 53]. To beter undestend adult brain mechanisms, this in vitro 2D model has been largely accepted by the scientific community and used to investigate well-connected and mature neuronal physiology [54–56]. By using a combined approach of histological and transcriptomic analyses, we revealed a modified neuronal morphology and network integrity, accompanied by elevated apoptosis levels after exposure to simulated microgravity and/or irradiation, which is indicative of CNS risks posed to the astronaut during space flight missions.

## Materials and Methods

### Primary Neural Cultures and the Neuronal Network Model

Primary neural cultures were initiated from the brain cortex of 17 day-old mouse fetuses as described in [26]. Cells were seeded onto poly-D-lysine coated 4-well plates or 12.5 cm^2^ flasks (Thermo Scientific, Aalst, Belgium) at a density of 50,000 cells per cm^2^, and were cultured for 10 days to obtain a dense neuronal network. After five days of culture, 2/3 of the medium was replaced by fresh medium every two days until simulated space condition exposure. All animal experiments were carried out in strict accordance with the recommendations of the Guide for the Care and Use of Laboratory Animals of the National Institutes of Health. The protocol was approved by the Belgian Nuclear Research Centre (SCK•CEN, Mol, Belgium) and the Flemish Institute for Technological Research (VITO, Geel, Belgium) joint Ethical Committee on Laboratory Animal Experiments (Permit number: 08–001). All required efforts were made to minimize suffering.

To study morphology, viability and gene expression changes, all 4-well plates (54 for acute irradiation or 24 for chronic irradiation) were filled with complete neurobasal medium at day 9 of culture (9^th^ DIV) and sealed with sterile parafilm. At 10 DIV, three replicates for each condition were mounted on a desktop RPM (Dutch Space) [57] to simulate microgravity. At this stage, the purity of neuron cultures is higher than 90%. This results was estimated by immunofluorescence analysis on βIII-tubulin positive cells and DAPI staining.

### Simulated Microgravity Exposure after Acute X-Irradiation

**Fig 1:**
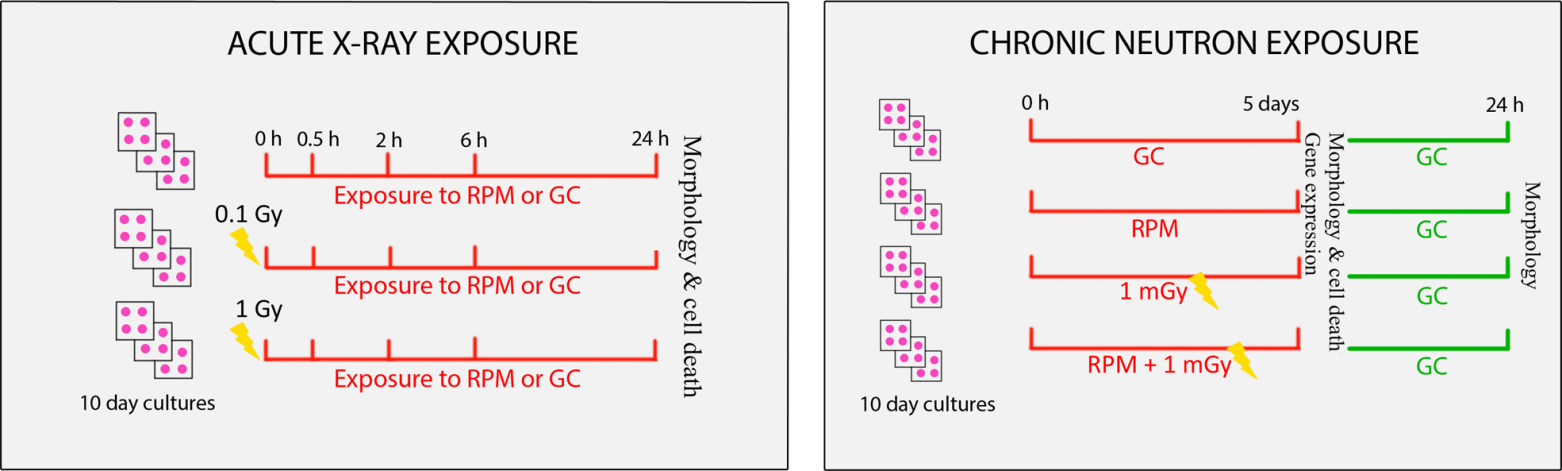
Schematic overview of the experimental set-up. RPM = random positioning machine, GC = ground condition, Gy = Gray.

In the acute exposure experiments (Fig 1A), 10 DIV neural cultures were first irradiated with X-rays, followed by simulated microgravity exposure. Cell cultures were irradiated at room temperature with 250 kV-15 mA, 1 mm Cu-filtered X-rays (Pentak HF420 RX machine), delivered at 5 mGy/sec. The Farmer 2570-EMI dosimeter was under the control of the Intercomparison Committee for Dosimetry. Cells were either non-exposed, exposed to a low dose of 0.1 Gy, or exposed to a high dose of 1.0 Gy of X-rays. Immediately after irradiation, half of the samples were placed on the RPM for simulated microgravity and the other half was kept in ground conditions as controls, both at 37°C and 5% CO_2_. Samples were analyses after 0.5 h, 2 h, 6 h or 24 h of simulated microgravity. Three replicates were used for each experimental condition.

### Simulated Microgravity Exposure during Neutron Irradiation

In the chronic exposure experiments (Fig 1B), 10 DIV neural cultures were irradiated with neutrons during RPM treatment. We used Californium-252 as source of neutrons and 2% of primary and secondary γ-rays, obtaining a final dose rate of 0.2 mGy/d to simulate space radiation [18, 58]. Additionally, the 98% neutron produced by Californium-252 has a quality factor (QF) of 10 [59]; consequently, the equivalent dose rate was 2 mSv per day. A total of four experimental conditions were used: (1) control cultures, (2) cultures exposed for five days to simulated microgravity, (3) cultures exposed for five days to neutrons at a dose rate of 0.2 mGy/d in ground conditions, and (4) cultures exposed for five days to neutrons at a dose rate of 0.2 mGy/d while exposed to simulated microgravity. Samples were analyzed immediately (0 h) and 24 h after exposure. Three replicates were used for each experimental condition.

### Immunohistochemistry and Image Analysis

All cell cultures were fixed with 4% paraformaldehyde for subsequent immunohistochemical analysis. Immunofluorescent stainings were performed using the monoclonal mouse-anti-βIII-tubulin primary antibody (Sigma-Aldrich, T5076, 1/100, Belgium) in combination with the fluorescein isothiocyanate (FITC)-conjugated anti-mouse IgG secondary antibody (Sigma-Aldrich, F2012, 1/200). Nuclei were stained with Hoechst (Sigma-Aldrich, B2883, 1/400).

Twenty-five mosaic regions of 2 by 2 images were acquired with a Nikon Eclipse Ti (automated inverted wide-field epifluorescence microscope) equipped with a 40x magnification (40x / 0.75) dry objective and a Nikon DS-Qi1Mc camera controlled by NIS-Elements software. Post-acquisition, images were compressed in a 2D in focus image by the Extended Depth of Focus (EDF) NIS-Elements module [49].

The neuronal network image processing analyses were performed using the MorphoNeuroNet, a home-made tool for ImageJ (Rasband, W.S., N.I.H, USA, http://rsb.info.nih.gov/ij/) [26, 49]. Thereafter, the average neurite area and length as well as soma morphology was determined and normalized per cell.

### Apoptosis Measurements

To quantitatively estimate the percentage of cell death, an Annexin V (Ann V)—propidium iodide (PI) assay was applied on adherent neurons using the Ann V-FITC apoptosis detection kit II (eBioscience, BMS500FI/300CE, Belgium), combined with Hoechst fluorescent staining to visualize all nuclei. The AnnV-FITC^-^/PI^-^/Hoechst^+^ (AnnV^-^-PI^-^) population was considered as normal healthy cells, while the AnnV-FITC^+^/PI^-^/Hoechst^+^ (AnnV^+^-PI^-^) and Ann V-FITC^+^/PI^+^/Hoechst^+^ (AnnV^+^-PI^+^) cells were considered early apoptotic and late apoptotic/necrotic, respectively. Non-neuronal cells with small nuclei and condensed chromatin, usually negative for βIII-tubulin, were not considered for analysis. Twenty-five images were acquired with a Nikon Eclipse Ti using a 20x objective and 500±150 cells per condition were taken into account.

Cell death was also determined by analyzing the degree of chromatin condensation and nuclear fragmentation, being a characteristic of apoptotic cells [60–62]. Nuclear fragmentation was defined by a high fluorescence intensity (>50% of saturated nuclear pixels) within a single nucleus [49]. Fragmented nuclei were counted as a percentage of the total number of nuclei.

### RNA Extraction

RNA was extracted from *in vitro* neuron cultures using the Qiagen AllPrep DNA/RNA/Protein Mini Kit according to the manufacturer’s instructions. Concentration and purity of RNA were assessed using the Nanodrop spectrophotometer (Thermo Scientific, USA) while RNA integrity was determined using the RNA Integrity Number (RIN) (Agilent’s lab-on-chip Bioanalyzer 2100, Agilent Technologies, USA). All samples had a RIN number above 8.0 and were used for further processing.

### Gene Expression Analysis

Microarrays were prepared as described previously [63]. Samples were hybridized onto Affymetrix Mouse Gene 1.0 ST Array Chips (Affymetrix, Santa Clara, USA), and gene expression profiles were analysed using Partek software (Partek Genomic Suite 6.6, Partek Inc. St-Louis, MO, USA). Quality control was performed according to Affymetrix instructions, all experimental conditions were scanned in one batch and each condition was repeated in triplicate. A two-way ANOVA analysis was used to assess differential gene expression between the different conditions, using condition as experimental factor. P-values were adjusted for multiple corrections using false discovery rate (FDR) as described by the Benjamini and Hochberg procedure [64], and genes were considered differentially expressed when FDR ≤ 0.05 and 1.3 ≤ fold-change ≤ -1.3. Differentially expressed genes were subsequently examined for gene ontology enrichment with the GOrilla tool [65], using a p-value of <0.001 and two unranked lists of genes (target list: differentially expressed genes, background list: genes expressed above background in at least 30% of all samples). To remove redundant Gene Ontology terms, results were visualised in REVIGO [66] with default settings.

### Reverse Transcriptase Quantitative PCR (RT-qPCR)

cDNA was synthesized using the Taqman Reverse Transcription kit (Applied Biosystems, California, USA) starting from 1 μg of total RNA per reaction and according to the manufacturer's instructions. RT-qPCR was performed using the MESA GREEN qPCR MasterMix Plus for SYBR assay I Low ROX (Eurogentec, Seraing, Belgium). Reactions were run in an Applied Biosystems 7500 Fast real-time PCR instrument (Applied Biosystems) with an initial hold cycle of 5 min at 95°C followed by 40 cycles of denaturation for 3 s at 95°C and primer annealing/elongation for 45 s at 60°C. Afterwards, a melting curve was performed and the reaction efficiencies for each of the tested primer pairs was assessed. Reaction efficiencies were used for relative quantification using the method as described by Pfaffl [67], and *Polr2a* and *Gapdh* were used as internal reference genes.

Primer sequences used included (from 5' to 3'):

FW_Slit2: GAG-CTG-GAA-AGA-CTG-CGT-TTA

RV_Slit2: CCT-TCC-TTG-GAA-TTG-CTT-GA

FW_Slc17a6: CTG-CAA-AGC-ATC-CTA-CCA-TTA-CAG

RV_Slc17a6: GTA- GAC-GGG-CAT-GGA-TGT-G

FW_Gabrb2: GCT-GCT-AAT-GCC-AAC-AAT-GAG

RV_Gabrb2: CCA-AGT-CCC-ATT-ACT-GCT-TCG

### Statistics

For morphological measurements and the assessment of apoptosis, a paired *t*-test (Graph Pad Software Inc., San Diego, USA) was applied and a *p*-value <0.05 was considered statistically significant.

## Results

### Neuronal Morphology Is Affected by Acute X-ray Exposure and Simulated Microgravity

To assess morphological consequences of simulated microgravity and acute X-ray irradiation on well-connected neurons *in vitro*, we determined the average neurite length and area as well as the soma area on βIII-tubulin stained neurons.

**Fig 2:**
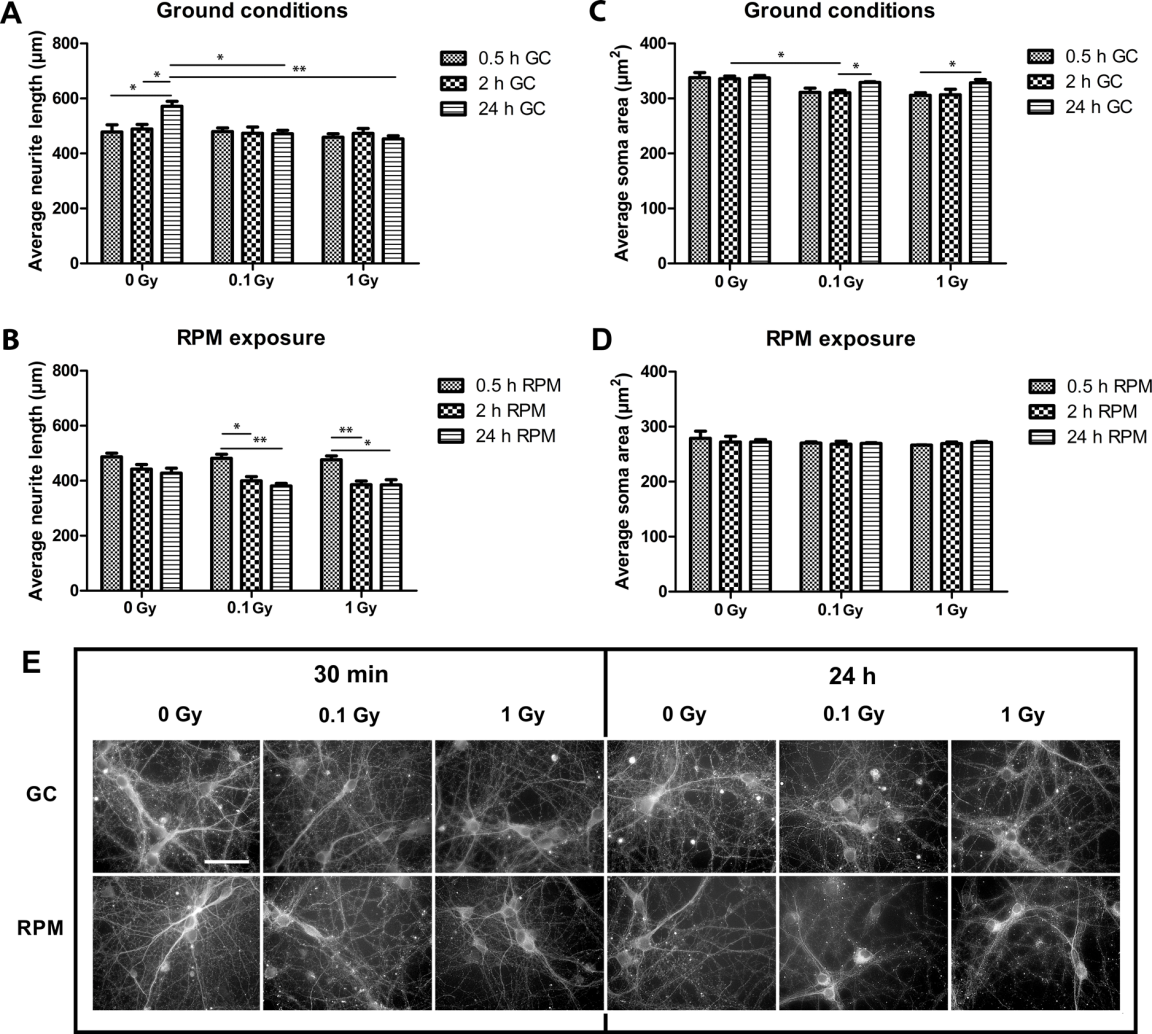
Neural morphology is altered after exposure to simulated microgravity and acute X-ray irradiation. **(A**) Morphometric analyses on βIII tubulin stained neurons in ground conditions revealed a significant effect of acute X-radiation on the average neurite length. In particular, irradiated neurons showed a stalling in neurite outgrowth within 24 h of culture. **(B)** Neurons co-exposed to RPM and irradiation showed a strong reduction in neurite length between 0.5 and 2 h of exposure, with no further reduction within 24 h. (**C)** Analysis of the soma area in ground conditions showed a clear trend towards a reduced soma size in irradiated neurons after 0.5 and 2 h. **(D)** Under RPM conditions, the overall soma size was smaller than in ground conditions, but no differences were revealed between the different irradiation doses. **(F)** Fluorescent images of neuronal network cultures X-irradiated with 100 mGy and 1 mGy followed by ground condition (GC) or RPM exposure for 30 minutes (30’) and 1 day (24h). RPM = random positioning machine, GC = ground conditions, Gy = Gray. Values are represented as mean±SEM. N = 3, * p<0.05, ** p<0.01, *** p<0.001.

While control cells showed a significant neurite outgrowth over the 24-h period, neurite outgrowth was completely arrested in irradiated cells (Fig 2A). Contrarily, in irradiated neurons co-subjected to simulated microgravity, a retraction of the neurites was noted within 2 h, with no further outgrowth within 24 h (Fig 2B). Similar results were found for the average neurite area (data not shown). Thus, in general, a more pronounced retraction of neurites was observed under microgravity as compared to ground conditions at 2 h and 24 h. When analyzing the cell somata, a clear trend towards a reduced soma size was noted in irradiated neurons 0.5 h (0.1 Gy: p = 0.093, 1.0 Gy: p = 0.053) and 2 h (0.1 Gy: p = 0.021, 1.0 Gy: p = 0.085) as compared to non-irradiated neurons, which recovered after 24 h (Fig 2C). Exposure to simulated microgravity resulted in an overall reduction in soma area as compared to ground conditions (p<0.05, Fig 2C and 2D), but combined exposure did not cause an additional reduction in size (Fig 2D). Thus, while neurite length and soma size were differentially affected by our simulated space conditions, exposure to simulated microgravity had an enhanced negative effect on both parameters.

### Neuronal Survival Is Compromised after Acute X-Irradiation and Simulated Microgravity

**Fig 3:**
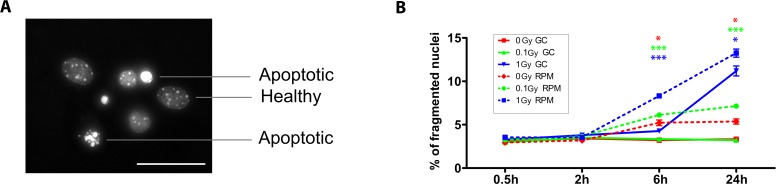
Neuronal cell death is increased after exposure to simulated microgravity and acute X-ray irradiation. **(A**) Representative image of fragmented/apoptotic *vs*. healthy nuclei in mature cultured neurons. **(B)** Analysis of the percentage of fragmented nuclei revealed a strong increase in apoptotic nuclei 6 h and 24 h after exposure to 1 Gy of acute X-rays (full blue line), as opposed to 0 and 0.1 Gy (full red and green line). Under RPM conditions, a similar dose-dependent trend was observed, with non-irradiated and 0.1 Gy irradiated neurons also showing an increased percentage of fragmented nuclei from 6 h onward (dotted lines). When kinetically comparing ground conditions with RPM exposure for the respective doses, a clear increase in the percentage of fragmented nuclei could be observed after 6 h and 24 h of RPM exposure for all doses. RPM = random positioning machine, GC = ground conditions, Gy = Gray. Values are represented as mean±SEM. N = 3, * p<0.05, *** p<0.001. Scale bar: 20 μm. Asterisks represent changes between RPM and ground conditions for the different radiation doses.

To reveal the effect of simulated space conditions on neuronal survival, we measured apoptosis in the different experimental conditions by analysing the percentage of fragmented nuclei (Fig 3A).

We observed that in ground conditions, exposure of cells to a low dose of X-rays (0.1 Gy) did not induce cell death. However, higher doses (1.0 Gy) led to an increased apoptosis starting at 6 h after exposure (p<0.05) with a strong increase after 24 h (p<0.01), when compared to non-exposed neurons (Fig 3B, solid lines). Furthermore, we found that RPM exposure resulted in an increased percentage of apoptotic nuclei after 6 h and 24 h, which was exacerbated in low- (0.1 Gy) and in high-dose (1.0 Gy) irradiated cells, as compared to the same radiation doses in ground conditions (Fig 3B). Thus, as for neuronal morphology, cell survival was shown to be particularly affected by RPM treatment.

### Simulated Microgravity and Chronic Neutron Irradiation Affect Neuronal Morphology

**Fig 4:**
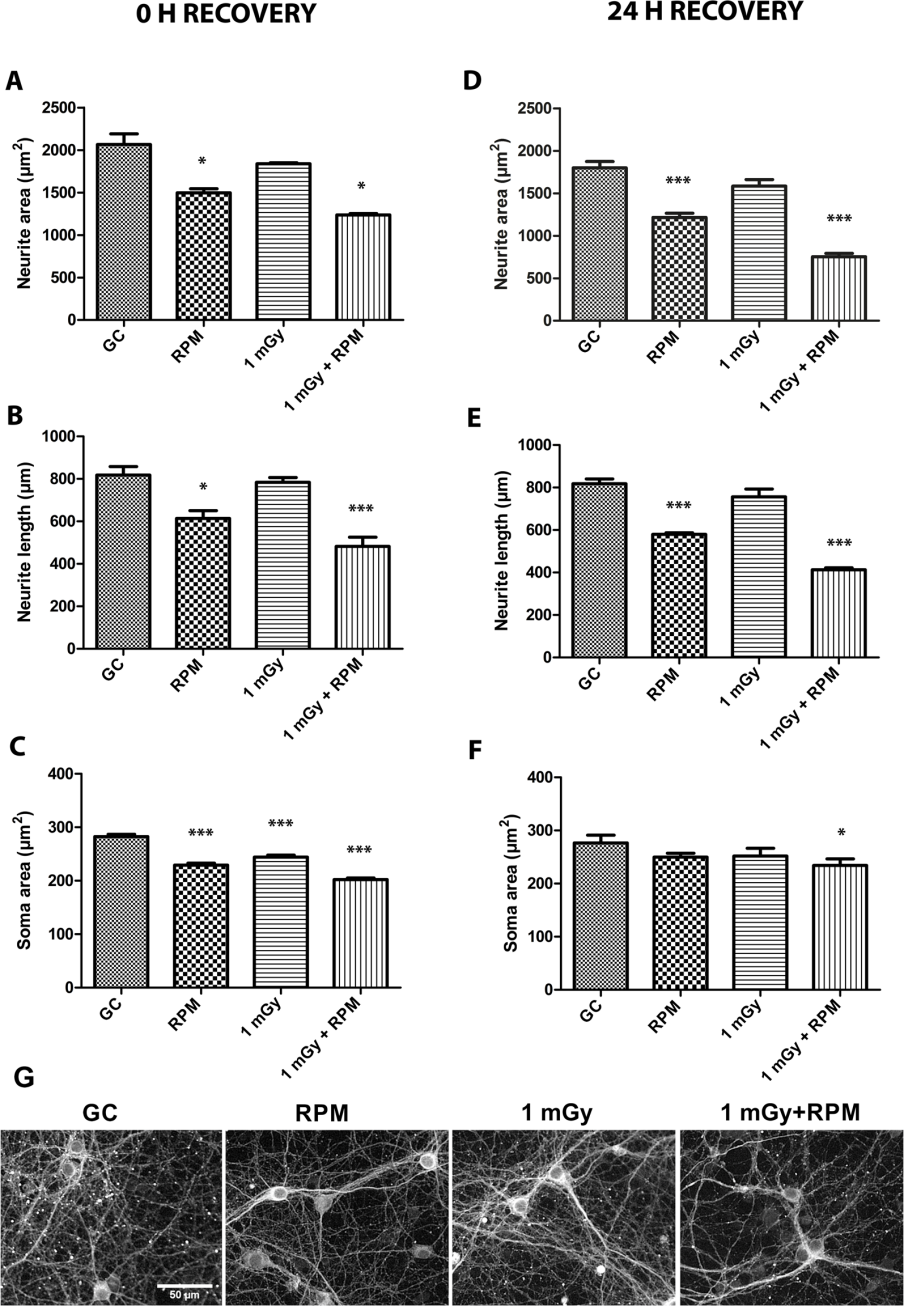
Neuronal morphology is altered after chronic RPM and irradiation exposure, and after ground recovery. **(A, B, C)** Morphometric analyses on βIII tubulin stained neurons immediately after 5-day chronic exposure conditions unveiled a significantly decreased neurite area **(A)** and neurite length **(B)** after RPM exposure and after combined RPM and radiation exposure. Analysis of the soma area revealed a strong decrease in soma area after all exposure conditions (RPM, irradiation and co-exposure) **(C)**. **(D, E, F)** Morphometric analyses after a 24 h ground recovery period showed a persistent and similar reduction in neurite area **(D)** and length **(E)** after radiation and/or RPM exposure, while the soma area was shown to be mostly recovered to control levels in RPM or radiation exposed neurons after 24 h recovery **(F)**. **(G)** Fluorescent images of neuronal network cultured exposed to ground conditions (GC), RPM, 1 mGy and 1 mGy combined to RPM. RPM = random positioning machine, GC = ground conditions, Gy = Gray. Values are represented as mean±SEM. N = 3, * p<0.05, *** p<0.001. Asterisks represent differences between RPM/irradiation groups and ground controls (GC).

To better simulate conditions as they occur during space flights or aboard the ISS, we exposed neuronal cultures to simulated microgravity for five days under continuous exposure to a source of Californium-252 emitting low doses of neutrons and γ-ray irradiation (Fig 1B). Morphometric analyses revealed a significant decrease in the neurite area after exposure to RPM but not after radiation exposure alone. Yet, the combination of both conditions had an additive negative effect on the neurite area (Fig 4A). Notably, we could reveal similar findings for the neurite length of these cells (Fig 4B). For the soma area, we found a significant reduction in cells exposed to radiation alone, while again the effect of RPM and particularly of co-exposure was most pronounced (Fig 4C).

Neuronal morphology of chronically exposed neurons was also evaluated in cultures recovered in ground conditions for 24 h (Fig 1B). Our data revealed a persistent decrease in neurite area (Fig 4D) and length (Fig 4E) in RPM and co-exposed neurons as compared to controls, which is well in line with what we observed immediately after chronic exposure conditions. Contrarily, after 24 h of recovery in ground conditions, the soma area was no longer reduced in neurons exposed to RPM or radiation alone, and the reduction in the co-exposed cultures, albeit significant, was now less clear compared to immediately after exposure (Fig 4F).

All together, these morphological data demonstrate a persistent reduction in neurite length and area, particularly after RPM and combined exposure. On the other hand, soma area reduction was less pronounced but was apparent in all conditions and seemed to recover during a 24 h period of re-adaptation to earth conditions.

### Chronic Microgravity and Neutron Exposure Results in a Synergistic Increase of Late Apoptotic Neurons

**Fig 5:**
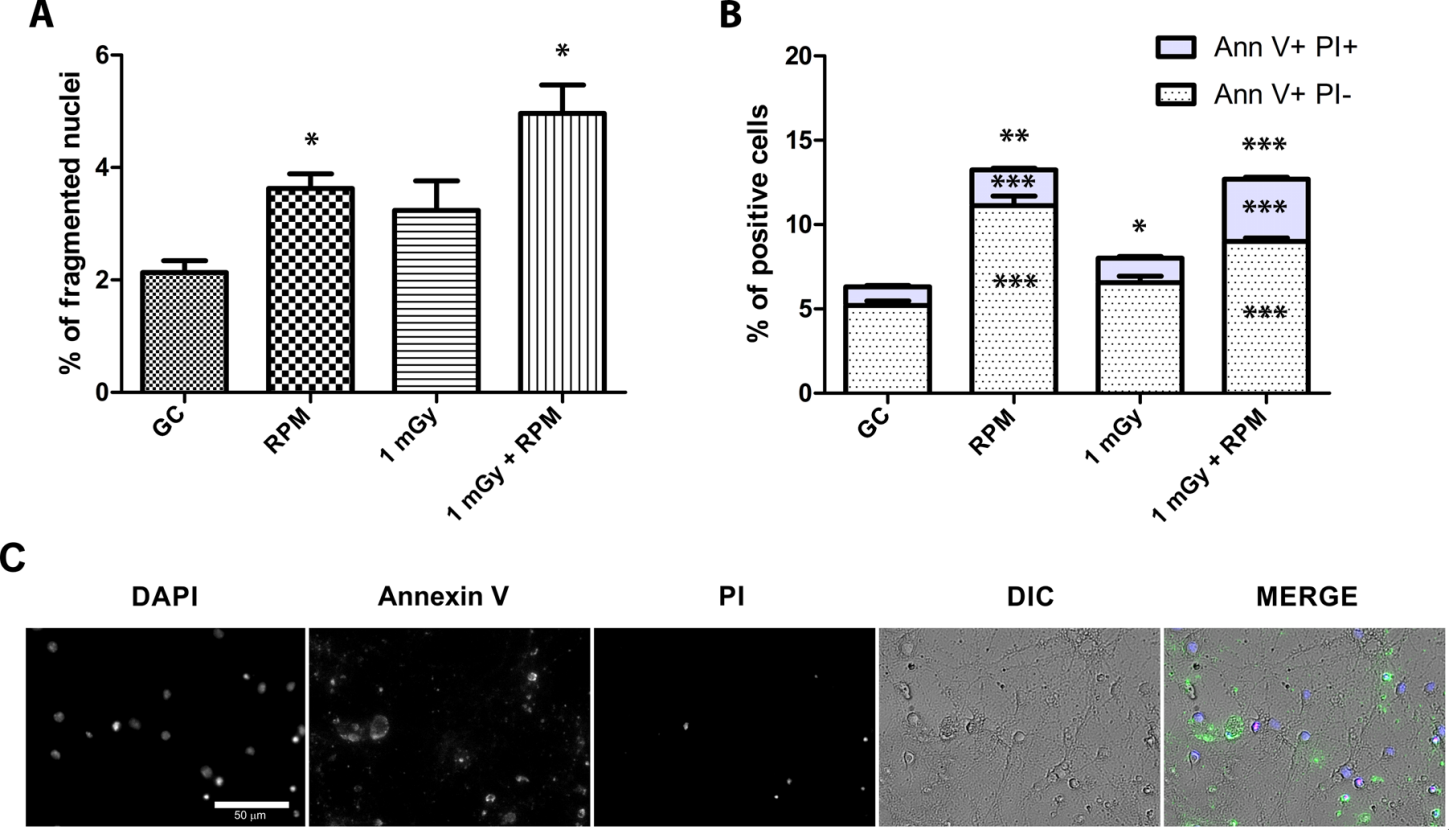
Co-exposure to chronic radiation and RPM results in a synergistically increased percentage of late-apoptotic neurons. **(A)** Analysis of the percentage of fragmented nuclei revealed a significant increase in apoptosis after RPM and combined exposure, but not after chronic exposure to radiation alone. **(B)** An Annexin V/PI apoptosis assay unveiled an increase in the total number of apoptotic cells (AnnV^+^/PI^+^ + AnnV^+^/PI^-^) in all conditions. However, when separately investigating early and late apoptosis, AnnV^+^/PI^+^ and AnnV^+^/PI^-^ labeled neurons were increased only after RPM and after combined exposure. Finally, only AnnV^+^/PI^+^ labeled neurons, representing late apoptotic neurons, were shown to be synergistically increased after combined exposure. **(C)** Nuclei, Annexin V, propidium iodide (PI), differential interference contrast (DIC) and merge representative images of neuron culture exposed to ionizing radiation and RPM. GC = Ground controls, RPM = random positioning machine, Gy = Gray. Values are represented as mean±SEM. N = 3, * p<0.05, ** p<0.01, *** p<0.001. Asterisks represent differences with non-exposed controls (GC).

To determine neuronal cell death after five days of chronic exposure to simulated microgravity and/or neutron irradiation, we analysed the percentage of fragmented nuclei showing condensed chromatin in respect to all nuclei, as well as the percentage of early and late apoptotic neurons by performing an *in situ* Ann-V assay. Comparable to the effects on neurite length and area, we noted an increased percentage of apoptotic cells after RPM exposure and after combined exposure to RPM and 1 mGy, while irradiation alone did not increase the proportion of fragmented nuclei, corresponding to late apoptosis (Fig 5A). To distinguish cells in early or late apoptosis, the AnnV-PI staining assay was performed, revealing an increase in the total number of apoptotic neurons (both early and late) after five days of exposure to irradiation, microgravity or both (Fig 5B). When analyzing early and late apoptotic neurons separately, no differences were found in neurons chronically exposed to radiation, whereas RPM exposure induced a 2-fold increase in early and late apoptotic neurons (Fig 5B). In case of combined exposure, the increase in the total number of apoptotic neurons was similar to RPM conditions. Yet, the relative increase in the percentage of late apoptotic/necrotic cells was much more pronounced (Fig 5B), which indeed correlates to the synergistic increase in fragmented nuclei and condensed chromatin in co-exposed neurons as shown in Fig 5A.

### Genetic Evidence for Changes in Neurite Extension and Synaptic Transmission in Chronically Exposed Neurons

**Fig 6:**
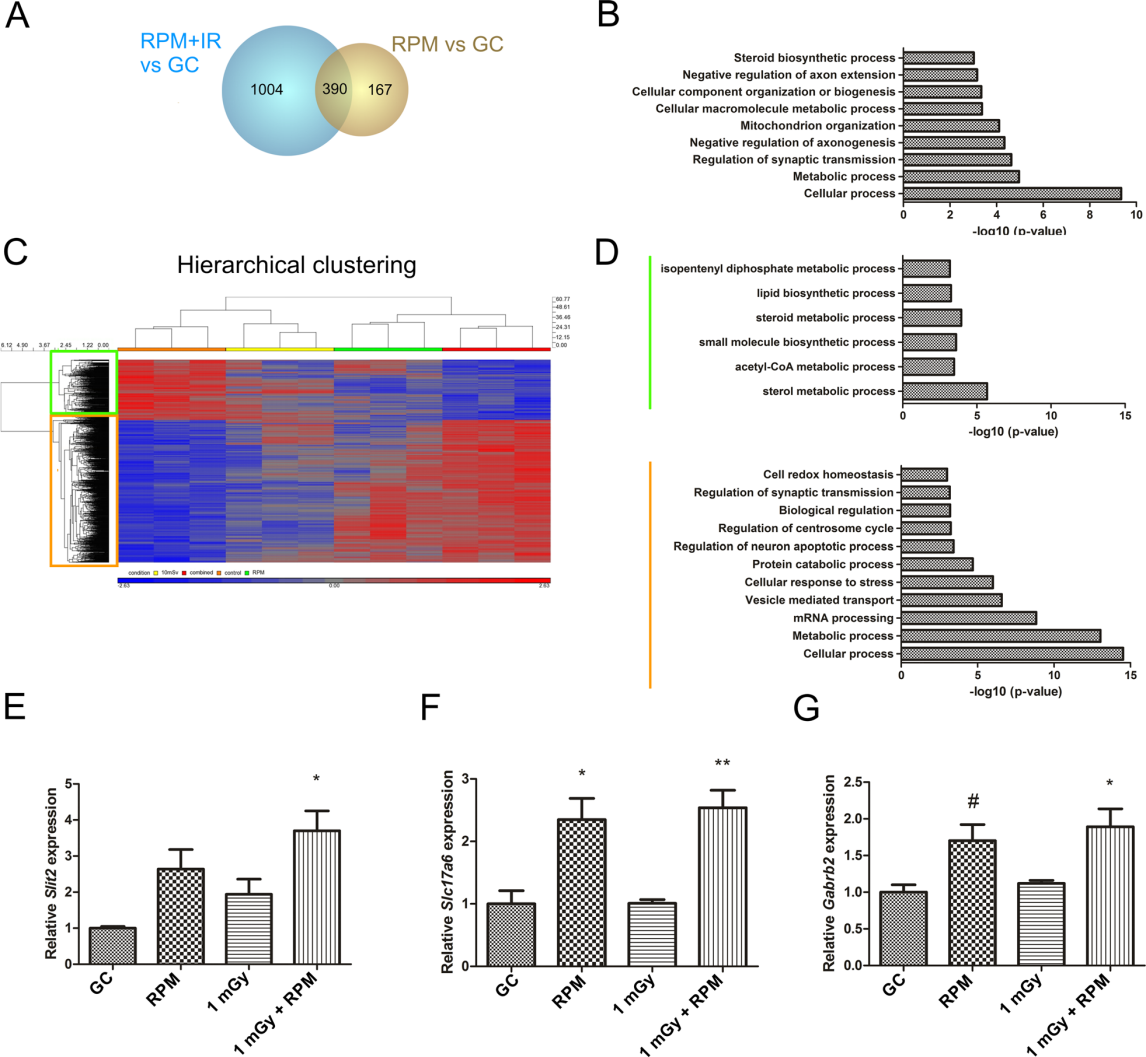
Gene expression and gene ontology enrichment analysis of neurons chronically exposed to RPM and irradiation. **(A)** A microarray analysis revealed differentially expressed genes in mature neurons immediately after chronic exposure to simulated space conditions (1394 genes in co-exposed neurons, 557 genes in RPM exposed neurons) as compared to non-exposed control neurons. Of those, 390 common genes were detected. No differential genes could be detected in irradiated neurons as compared to control cells. **(B)** A gene ontology enrichment analysis showed an enrichment of the 390 overlapping genes in pathways related to e.g. synaptic transmission, axonogenesis or biogenesis. **(C)** A hierarchical clustering of all genes differentially expressed between co-exposed and non-exposed neurons (1394) showed a large gene cluster that was upregulated in co-exposed neurons (yellow square), and a smaller cluster of downregulated genes (green square). **(D)** A gene ontology enrichment analysis of the downregulated gene cluster showed the involvement of few processes, which were not linked to the observed histological phenotype, whereas a similar analysis of the upregulated gene cluster predicted the involvement of e.g. synaptic, apoptotic, cell redox or metabolic processes. **(E-G)** qPCR experiments for neurite guidance and synaptic transmission genes confirmed an upregulation of the repulsive gene *Slit2* in co-exposed neurons (E), as well as an upregulation in excitatory synapses in RPM and co-exposed neurons (*Slc17a6*) (F) and in inhibitory synapses in RPM (borderline significant) and co-exposed neurons (G). RPM = random positioning machine, IR = irradiation, GC = ground condition. Values are represented as mean±SEM. N = 3, # p = 0.05, * p<0.05, ** p<0.01. Asterisks represent differences with non-exposed controls (GC).

In order to identify genes and pathways affected by simulated space conditions and possibly responsible for the observed phenotypic changes, we performed a microarray analysis on mature neurons that were either non-exposed, exposed to RPM or chronic neutron irradiation, or both. Immediately after chronic exposure, 557 and 1394 genes were found to be differentially expressed after RPM and combined exposure respectively, as compared to non-exposed neurons (Fig 6A). Surprisingly, whereas we could identify 71 differential genes between 1 mGy and RPM exposed neurons, and 41 differential genes between 1 mGy and co-exposed neurons, chronic low dose radiation did not cause any changes in gene expression as compared to the control condition. This correlates well with our previously described morphological and survival data, in which single chronic radiation exposure did not induce an overall effect in cultured mature neurons.

Our analysis revealed a very significant overlap of differentially expressed genes after exposure to RPM and exposure to combined conditions (390 genes, Fig 6A). When considering these overlapping genes, we discovered the involvement in e.g. chemotaxis or synaptic transmission, thereby hinting towards changes in network remodelling after RPM exposure and combined chronic space simulation. To substantiate this, we performed a gene ontology enrichment analysis on this set of genes. With this, we could identify several significantly affected pathways involved in e.g. regulation of axon outgrowth and regulation of synaptic transmission (see Fig 6B for a non-exhaustive list). Furthermore, unsupervised hierarchical clustering of the 1394 genes differentially expressed in co-exposed neurons revealed that the majority of these genes (67%) were upregulated after the treatment (Fig 6C). Gene ontology enrichment of the small cluster of downregulated genes showed only a few gene sets to be involved (Fig 6D, green bar), at first sight not related to the observed neuronal phenotype described before. Gene ontology enrichment of the upregulated genes, on the other hand, identified a number of related gene sets (see orange bar in Fig 6D for a non-exhaustive list), including those involved in regulation of neuron apoptotic processes and synaptic transmission.

To verify a reduced neurite length and a possibly altered neurite guidance or synaptic communication as was proposed from our gene expression analysis, we performed qRT-PCR experiments for genes involved in these pathways. This indeed showed an increase in the repulsive molecule *Slit2*, immediately after five days of combined exposure to chronic neutron irradiation and microgravity, as compared to ground control neurons (Fig 6E). Further, the expression of vesicular glutamate transporter 2 (*Slc17a6* or *Vglut2*), a regulator of excitatory glutamate availability, and the GABA receptor B2 (*Gabrb2*), an inhibitory postsynaptic GABA receptor, were significantly increased both after exposure to RPM as well as in co-exposed neurons (Fig 6F and 6G). This thus clearly indicates changes in synaptic transmission and communication in neurons subjected to simulated space conditions.

In general, we were able to strengthen our morphological and gene expression data, and propose a negative effect of chronic RPM and combined RPM/irradiation exposure on neurite outgrowth, cell communication and cell survival in mature neuronal networks.

## Discussion

In this study we evaluated the effects of simulated space conditions, i.e. exposure to ionizing radiation combined with a simulation of weightlessness, on well-connected mature cortical neuron cultures. In first instance, neurons were subjected to acute X-irradiation followed by a 24 h period of simulated microgravity. Secondly, in order to better reproduce space conditions aboard the ISS, chronic exposure to Californium-252, a source of neutrons and γ-rays, was used over a five-day course (0.2 mGy/d approximates the maximal dose rate achievable during ISS missions) in combination with microgravity exposure. We then evaluated the effect of both simulated space conditions on neuronal morphology as well as on cell death events. In addition, earth re-adaptation was simulated by implementing a recovery period of 24 h, in order to analyze a potential recovery of neuronal morphology [26]. Finally, gene expression experiments were performed as a useful tool to discover the underlying molecules and pathways implicated in the histological observations.

Morphological analyses of βIII tubulin-stained neurons revealed a strong neurite retraction particularly after RPM exposure and co-exposure to RPM and radiation, which was the case for both acute and chronic exposure paradigms. This observation corresponds well to previously published data showing a disturbed neuronal morphology of mature cultured neurons exposed for 1 h and 24 h to RPM conditions [26]. Notably, however, a long-term follow-up of these RPM-exposed neurons indicated an attenuation of the induced defects after 10 days of exposure, suggestive of cell adaptation to the new gravitational field [26], and thus warranting a longer follow-up of co-exposed neurons in future set-ups. When considering acute exposure, in which low and high doses of radiation were administered acutely and neurons were monitored over time, we could show that irradiation alone merely resulted in stalling of neurite outgrowth for at least 24 h. This milder effect of irradiation as compared to RPM on neurite outgrowth was even more evident after chronic (co-)exposure, where we found no difference in neurite length and area in neurons solely exposed to irradiation for five consecutive days, whereas a strongly reduced neurite area and length was noted in RPM and co-exposed neurons. This clearly suggests a different effect of RPM and irradiation on neurite extension and thus on neuronal outgrowth. In this respect, it is important to note that gene expression analyses on chronically exposed neurons did not reveal any differentially expressed genes after chronic exposure to radiation, whereas many genes were differentially expressed in RPM and co-exposed neurons as opposed to non-exposed neurons. In all, the obvious morphological defects that occur after a change in gravitational field combined with exposure to ionizing radiation might be attributable to cytoskeletal changes, which can also be suggested from our gene expression data. This would be in line with other studies revealing cytoskeletal changes due to altered gravity, as shown by microfilament and microtubule changes [8, 26, 68, 69], and possibly related to a cell surface reduction [7].

The observed reduction in neurite outgrowth in neurons exposed to microgravity and/or combined with irradiation might also imply an improper network formation and synaptic communication. Therefore, we analyzed gene expression profiles after a chronic exposure to radiation and/or RPM, with the purpose of identifying related gene sets. Interestingly, this unveiled changes in expression of genes related to synaptic transmission, chemotaxis and cellular organization, from which some were validated by RT-qPCR. Somewhat counterintuitively, we noted an increased expression of excitatory and inhibitory synaptic genes. A compensatory increase in neurotransmitter transport and neuron excitability after chronic exposure to stress stimuli might be at play, although this increased expression does not necessarily imply an improved signal transduction in the brain. For example, a compensatory upregulation of GABA receptors was detected in schizophrenic patients with reduced numbers of inhibitory neurons and was suggested to highly contribute to the pathophysiology of the disease [70]. Further, an increased VGluT expression might be intrinsically linked to excitotoxic neurodegeneration and associated neuronal pathologies [71]. In light of these results, a closer look at synaptogenesis and possible degenerative events will be key to better understand neuronal communication and wiring in brains exposed to space conditions. In view of this, a recent study by Demertzi *et al*. discovered changes in brain connectivity, vestibular ataxia as well as changes in motor coordination in a cosmonaut during his mission aboard the International Space Station [72]. A defective brain wiring is further corroborated in mice exposed to HZE particles, in which an altered behavior, associated with changes in medial prefrontal cortex and hippocampus connectivity, was detected. A concomitant decrease in dendritic arborization and dendritic spine density was observed in these animals, which further establishes the functional deficit resulting from cosmic rays [44]. Notably, since changes in neuron morphology and synaptogenesis were particularly evident in neurons co-exposed to space conditions in our study, the results shown by Parihar et al. [44] might even be underestimated and should include a combined exposure to simulated microgravity to reveal the true impact on the CNS.

Based on previous reports linking a defective synaptogenesis and synapse density to a reduced soma size [73, 74], we next envisioned to analyze the soma area in our set-up. Exposure to simulated microgravity alone and in combination with acute or chronic irradiation indeed resulted in a fast reduction of the soma size already after 0.5 h, which persisted in chronically exposed neurons. This is in accordance with our previous results showing a decrease in the soma size after 1 h and 24 h of RPM exposure, and persisting until 10 days [26]. Surprisingly, whereas acute X-ray exposure had no influence on the soma size, chronic low-dose neutron radiation resulted in a reduced soma size. In keeping with the fact that synaptogenesis genes were not altered and neurite area and length were not affected in these chronically irradiated neurons, a closer look at the somata in these cells thus deems necessary to correctly interpret our finding. Nevertheless, our data imply that a short, acute, exposure to radiation might not be sufficient to induce differences in soma morphology, or that a difference in the chosen radiation paradigm might differently affect the cell body.

In an attempt to simulate the recovery of exposed neurons to earth conditions after a space flight, we subjected neurons to ground conditions for 24 h and re-assessed neuronal morphology parameters. From this, a persistently aberrant neurite area and neurite length was unveiled, again especially apparent for RPM and co-exposed neurons. Of importance, this finding is in accordance with previous findings [26], showing a decrease in the neurite area and length during the first 24 h of ground recovery after RPM exposure, and showing a higher recovery potential of cell somata as compared to neurites. As such, this knowledge on early CNS events that occur after re-introduction to ground conditions are a good indicator of recovery potential, and can serve to further investigate long-lasting changes in neuronal morphology in astronauts that return from a space flight.

Apart from neuronal morphology, we also assessed cell survival in neurons exposed to RPM and/or irradiation. Analogous to the neurite outgrowth profile, we showed an increased apoptosis in RPM and co-exposed neurons, either acutely or chronically, as compared to controls, whereas irradiation alone–at least for the low dose of 0.1 Gy–did not generally induce apoptosis. Intriguingly, the fact that we did not observe apoptosis after 2 h of RPM is in sharp contrast to previously obtained data in which RPM-induced apoptosis was already apparent after 1 h exposure [26]. This discrepancy might possibly be explained by a different analysis method, i.e. assessment of the percentage of fragmented nuclei in our current analysis *vs*. an AnnV-PI analysis in the former study. Since fragmented nuclei only account for late apoptotic cells, the increase in apoptosis within the first 24 h of RPM exposure might thus be underestimated in our study, which hinders a correct comparison between both studies. Yet, noteworthy, we distinguished between early and late apoptotic cells after a 5-day chronic exposure to weightlessness and neutron irradiation. From this, we showed a significant increase in early as well as late apoptotic cells in RPM and co-exposed neurons, while we could reveal a synergistic effect of combined space conditions on the amount of late apoptotic cells. A sound explanation for this synergistic elevation in late apoptotic cells is still lacking at the moment, but might be in accordance with a faster induction of the cell death pathway when cells are simultaneously exposed to both space conditions. Importantly, a gene ontology analysis of differentially expressed genes between RPM/co-exposed neurons and non-exposed controls could further confirm a compromised cell survival, and showed their involvement in e.g. 'cellular response to stress' and 'regulation of cell death' after a five-day chronic exposure period.

With regard to the effects of combined space conditions on cells or tissues (e.g. immune cells, germ cells or bone tissue), space life investigators proposed three different theories: additive [47, 75, 76], antithetical [46] or non-correlative [77]. In this study, we unveiled an additive effect of the combined chronic treatment on both the neurite and soma area, whereas the neurite length as well as the number of late apoptotic neurons were shown to be synergistically affected. Thus, while the overall neuronal morphology is compromised after combined space conditions, neurite outgrowth and survival seem to be particularly prone to the space environment, raising concerns with respect to neuronal connectivity and communication in astronauts while aboard a space craft. In all, by taking into account both space conditions, we set the stage for future experiments using for example additional time points or *in vivo* approaches, which will undoubtedly lead to a more complete understanding of processes occurring within the central nervous system during an astronaut's space flight and during recovery back on earth.
